# Population based haematology reference ranges for old people in rural South-West Uganda

**DOI:** 10.1186/s13104-016-2217-x

**Published:** 2016-09-07

**Authors:** Joseph O. Mugisha, Janet Seeley, Hannah Kuper

**Affiliations:** 1 MRC/UVRI Uganda Research Unit on AIDS, P.O. Box 49, Entebbe, Uganda; 2 London School of Hygiene and Tropical Medicine, London, UK; 3 School of International Development, University of East Anglia, Norwich, UK

**Keywords:** Old people, Uganda, Africa, Haematology reference ranges

## Abstract

**Background:**

Haematology reference values are needed to interpret haematology results and make clinical decisions, but these have not been established for old people in sub-Saharan Africa. The objective of this study was to establish haematology reference values for people aged 50 years and above in Uganda, to compare the haematology reference values for those aged 65 years and over with those less than 65 years and to compare these haematology reference values with established haematology reference values for old people from high income countries.

**Methods:**

A total of 1449 people aged 50 years and above were recruited from the Medical Research Council/Uganda Virus Research Institute general population cohort between January 2012 and January 2013 (response rate 72.3 %). From the blood samples collected, we did haematology, HIV testing and malaria tests. We also obtained stool samples and tested them for hookworm infection. Questionnaire data were obtained through interviews. In the analysis, we excluded those with HIV infection, malaria infection, hookworm infection and those not feeling well at the time of recruitment. Medians and reference ranges for 12 haematology parameters were determined, based on the Clinical Laboratory and Standards institute’s guidelines.

**Results:**

In total, 903 people aged 50 years and above were included in the analysis with the majority 545 (60.3 %) being female. Men had significant difference in median haemoglobin, haematocrit, erythrocytes counts and white blood cells counts, which were higher than those of women. Women had significant difference in mean platelet counts and neutrophil percentages which were higher than those of men. Comparing those aged 65+ and those aged less than 65 years, the following parameters were significantly lower in those aged above 65 years: haemoglobin, haematocrit, erythrocytes counts, platelets and mean corpuscular volume. Compared to the reference intervals from old people in high income countries, all the haematology parameters from our study population were low.

**Conclusion:**

The differences between haematology reference ranges in old people compared to adults and the very old (65+) compared to those between 50 and 65 call for more population based studies using nationwide surveys to be carried out among old people in other study settings in Uganda and the rest of Africa to explore the differences in haematology reference ranges between these different age groups with a view of establishing whether there is need to have separate reference range for these different categories of old people.

## Background

Haematology reference values are important in identifying abnormal blood results and for guiding clinicians in the management of patients with disorders of the blood and other illnesses. In Uganda, like most other African countries that do not have locally established haematology reference values for haemoglobin and full blood counts, the reference values used for haematology parameters are those derived from data collected from white people of from high income countries [1, 2]. In addition, these haematology reference values from studies in white people are also used to make guidelines, such as those of the WHO, used in the definition of anaemia [3].

Haematology reference values derived from white people may be different from those derived from black people in Africa, as shown by studies conducted in Africa [1, 2, 4–6]. Generally, reference values for haemoglobin in black Africans are lower than those in white people while those for platelets are higher than those in white people. A number of factors have been suggested for these differences, including genetic variation, the higher prevalence of pathogens like malaria and hookworm in Africa, poorer nutritional status of individuals and altitude [7].

In addition to the question of whether reference values based on whites are generalisable to other populations, there is also debate on whether haemoglobin and other haematology reference values derived from young adults are suitable for old people [8]. For example regarding haemoglobin, a number of studies conducted in high income countries have shown that haemoglobin declines with age especially in males as compared to females [9–11]. It has been suggested that there may be some age- related conditions that may determine the reduction of haemoglobin, such as: reduction in the reserve of haematopoietic progenitors, decreased responsiveness of these progenitors to stimulatory growth factors and reduced erythropoietin production [12], which may contribute or result to lower reference ranges for haemoglobin in healthy older adults compared to healthy younger adults. Such age related differences in haematological reference ranges have been reported in high income countries [13, 14].

From our literature search, we did not come across any study from Uganda on haematology reference ranges for old people. We identified four studies that were conducted in Uganda on haematology and other laboratory reference ranges like CD4 cell counts and blood biochemistry [1, 4, 5, 15] in young adults. All these studies, except one, were conducted in preparation of HIV vaccine trials. These studies included very few old people (<2 %). More importantly, these studies did not test the study participants for some infections like malaria and hookworm infestations which are prevalent in Uganda and have been shown to be related to anaemia, so that the reference ranges derived from these studies may not reflect the actual reference ranges of healthy people within these populations. Studies done elsewhere in Africa to establish local haematology and biochemical reference values have also not targeted older people [2, 16–19] with the exception of one study conducted in Zimbabwe [20].

Since the population of older people is increasing in Africa [21] and research and care for older people infected with HIV/AIDS especially those on anti-retroviral therapy (ART) is gaining momentum [22, 23], it is necessary to establish local haematology reference ranges for older people in order to improve on the interpretation of haematology results for older people.

We used data from the anaemia survey that was conducted in older people in the General Population Cohort (GPC) in rural South West Uganda to create haematology reference ranges for old people including hemoglobin, white blood cell counts (WBC) totals and differentials, red blood cell counts (RBC), haematocrit (HCT), mean corpuscular volume (MCV), mean corpuscular hemoglobin (MCH) and platelets (PLT). We compared haematology reference ranges for people aged 50 to ≤65 years and those aged >65 years and compared these reference ranges with those reported from old people in high income countries [13, 14, 24, 25].

## Methods

### Study population

Study participants aged 50 years and above were recruited during the anaemia survey that was conducted in the General Population Cohort [26] round 23 between January 2012 and January 2013. Eligible participants were elder residents that gave consent to participate in the study from 25 study villages during the survey period, were willing to give a blood and stool sample and were willing to be interviewed. Participants were excluded if they were too ill to answer questions.

### Justification for inclusion of 50–59 age groups

There are many definitions that have been used to describe older people. However, there is no general agreement on the age at which a person becomes old, although generally they can refer to people aged 60 years and over [27]. In many developed countries, the reference age can be pushed up to 65, which is the age at which people become eligible for old age social security benefits [28]. In sub Saharan Africa, it is acceptable to use 50 years old as a cut off as previously defined by the minimum data set project [29] of the World Health Organisation, due to the low life expectancy in this region.

### The general population cohort

Details of the general population cohort have been described previously [30]. In brief, the GPC is a population-based cohort study of around 20,000 people living within the Kyamulibwa sub-county of Kalungu district in rural South-West Uganda. The cohort was established in 1989 by MRC/UVRI Uganda Research Unit on AIDS to describe trends in the prevalence and incidence of HIV infection and their determinants in the general population. The initial recruitment was conducted in 1989/1990.

Every year since 1989, annual house-to-house “rounds” of census and survey have been carried out until 2012 when the house to house survey method was changed to active participation by inviting and surveying residents at central hubs (a building rented for the purpose in the village) in each of the study villages.

Demographic, socio-medical and serological data are collected. Information regularly obtained includes data on fertility, mortality, migration, sexual behavior, perceptions of HIV infection and HIV status.

The GPC runs a clinic and a clinical laboratory at the Kyamulibwa field office where GPC participants can access free treatment. The clinic is run by two clinicians and is stocked with most of the essential drugs recommended by the Ministry of Health of the Uganda government.

In addition to conducting annual HIV sero surveys, the GPC also provides a population for recruitment for other studies including the rural clinical cohort aimed at understanding the clinical course of HIV disease in adults and improving treatment outcomes for HIV infected participants and social science research aimed at an in-depth understanding of social aspects of health, including research on the health and wellbeing of older people.

The study reference population has prevalent diseases like malaria, worm infestations especially hookworm, HIV/AIDS and a number of respiratory tract infections.

### Data collection for the anaemia survey

A questionnaire for the anaemia study was developed based on existing questions in the GPC survey questionnaire [26], the World Health Organisation study on global ageing and adult health [31] questionnaire and the cross cultural assessment of nutrition in older subjects questionnaire [32]. For this analysis, we collected questions specifically on the social demographic characteristics of study participants, alcohol and tobacco use, self-rated health at the time of the interviews, history of fever, use of drugs for malaria treatment in the past month prior to the interview, history of worm treatment in the past month prior to the interview and history of cancer or cancer treatment. The anaemia study questionnaire was translated into the local language and back translated into English and necessary adjustments made. The questionnaire was then pilot tested using 63 people belonging to the target age group. The final questionnaire was then programmed on ultra-mobile personal computers (UMPCs) which were used for data collection. All older people in the GPC were first enumerated and mobilized through community meetings, and home visits. Those that consented to participate in the anaemia survey were either interviewed from the village central hubs or were interviewed from the GPC study clinic. All interviews were conducted by trained interviewers from the GPC.

### Sample collection

Phlebotomy was performed by a nurse on one arm with the respondent in the seated position. Five milliliters of blood were drawn in an EDTA tube and inverted 5–6 times to ensure mixing with EDTA anticoagulant to prevent clotting. Blood samples were drawn between 8.30 a.m. and 2.30 p.m. each day. Participants did not undergo fasting prior to phlebotomy. Two consecutive stool samples were obtained in stool bottles which had been provided together with instructions a day prior to the interview. The samples were stored in cold boxes and transported on the same day to the Kyamulibwa clinic laboratory by an MRC/UVRI van.

### Haematology

A complete blood count with a differential was performed on the on the same day the sample was collected. The haematology was performed at the Kyamulibwa clinical laboratory using a Beckman Coulter A^C.T^ 5 diff CP Haematology analyser (Beckman Coulter, USA), following the manufacturer’s instructions.

### HIV testing

HIV testing was done using an algorithm for HIV-testing with three HIV-1 rapid tests as recommended by the Uganda Ministry of Health. The algorithm for HIV rapid testing consisted of an initial screening with the rapid test Determine HIV1/2 (Abbot Laboratories by Abbot Japan, CO LTD, Minato-Ku, Tokyo Japan). If the test result was negative the participant was given a diagnosis of HIV negative with no further rapid testing. If the test result was positive the sample was retested with the rapid test HIV 1/2 Stat-Pak (Chembio Diagnostics System 3661 Horseblock Road, Med Ford, New York 11763, USA). If both tests gave a positive result the participant was given a diagnosis of HIV positive with no further rapid testing. If the tests gave discordant results (one positive and the other negative), the sample was further evaluated with the rapid test Uni-Gold Recombinant HIV-1/2 (Trinity Biotech PLC, IDA Business Park, Bray, Cowicklow, Ireland). For those samples assessed by all three tests, two positive test results were interpreted as a positive diagnosis. If two of the three tests gave negative results then the participant was diagnosed as being negative for HIV.

### Malaria testing

Malaria was defined as having a positive blood slide for malaria parasites. Thick and thin, Giemsa-stained blood smears were prepared and microscopically examined for malaria parasite and species.

### Hookworm testing

Two slides per stool sample were prepared at the Kyamulibwa clinical laboratory and examined for hook worm ova using the Kato Katz method [33]. Results were classified based on WHO infection guidelines as light [less than 2000 eggs per g (EPG) of faeces], moderate (between 2000 and 4000 EPG) and heavy (above 4000 EPG).

### Quality control

All laboratory tests were performed by trained laboratory technologists following special operating procedures of the MRC/UVRI laboratories. External quality assurance for the MRC/UVRI laboratories was also carried out every year by National Health Laboratory Services (South Africa) and Royal College Pathologists Australia for the automated machines twice a year. In addition to these, laboratory audits were conducted on the MRC/UVRI laboratories twice a year by Clinical Laboratory services South Africa and Qualogy Ltd for good clinical laboratory practice annually. Controls for the haematology analyser were done on a daily basis using commercially manufactured COULTER A^C.T^5 diff control plus (Beckman Coulter, USA) and were classified as low, normal and high.

### Ethical considerations

Ethical clearance was obtained from London School of Hygiene and Tropical Medicine ethics committee, Uganda Virus Research Institute Research and Ethics Committee and the Uganda National Council for Science and Technology. We obtained informed consent from all the study participants and all participants requiring medical treatment were referred to the GPC study clinic and nearby government health centres.

### Data management and statistical methods

Questionnaire data were uploaded from the UMPCs onto a networked computer and automatically stored onto the server each evening at the MRC/UVRI field station. The following morning, the data were checked and uploaded onto the database. Laboratory data were double entered into access computer program by data entry officers at the field station. After data cleaning (looking for missing values, removing extreme values and wrong entries), questionnaire data were merged with laboratory data. Data analysis was performed using STATA 11 software (Stata Corp, College Park, TX, USA). In the analysis we excluded participants with malaria, hookworm, HIV/AIDS or those who reported that they were not feeling well at the time of the survey. Since most of the literature on haematology reference intervals has shown differences between men and women, we stratified the data by gender. We also stratified the data by age (comparing older people 50–65 years and those 65 years and above). All analysis for establishing the reference values were based on the CLSI (Clinical and Laboratory Standards Institute, 2008) [34].

We first performed a goodness of fit test. Since most of the parameters were not normally distributed according to the Shapiro–Wilk test for normality, we used a non-parametric method as a procedure for establishing reference values [34]. With respect to outliers, we used Dixons method [35]. Medians and reference values were obtained directly from the data and p values for the differences between men and women were estimated using the Mann–Whitney test. The reference values were calculated as the range between those values at the 2.5 and 97.5 % limits for the population. The reference values thus encompassed 95 % of our study population.

## Results

### Description of study population and exclusions

In total, questionnaire and haematology data were available for 1455 participants (72.3 % of the total population of older people within the GPC). More than half (58.2 %) of the participants interviewed were female (Table 1). After excluding those with hookworm infection (412), current malaria infection (41), those who were HIV positive (94), and those who were uncertain about their age (5) we had data for 903 older people (62.1 %) of which 545 (60.3 %) were female (Table 2).

**Table 1:** Description of study participants in the anaemia survey

	Males (N = 605)	Females (N = 844)
Age (years)		
50–59	257 (43 %)	344 (40.8 %)
60–69	155 (26 %)	291 (34.5 %)
70–79	138 (23 %)	149 (17.7 %)
80+	55 (9 %)	60 (7.1 %)
Marital status^a^		
Married	427 (70.7 %)	276 (32.7 %)
Divorced/separated	115 (19.0 %)	218 (25.9 %)
Widowed	53 (8.8 %)	346 (41.0 %)
Single (never married)	9 (1.5 %)	3 (0.4 %)
Education level		
None/incomplete primary	125 (20.7 %)	281 (33.3 %)
Primary	372 (61.5 %)	500 (59.2 %)
Secondary	90 (14.9 %)	45 (5.3 %)
Above secondary	18 (3.0 %)	18 (2.1 %)
SES score tertile^b^		
Low	186 (36.7 %)	253 (33.8 %)
Middle	175 (34.5 %)	294 (39.3 %)
High	146 (28.8 %)	201 (26.9 %)
Smoking		
Current smoker	188 (31.1 %)	36 (4.3 %)
Past smoker	153 (25.3 %)	31 (3.7 %)
Never smoked	264 (43.6 %)	777 (92.0 %)
Chewing tobacco^c^		
Current user	112 (18.5 %)	184 (21.8 %)
Past regular user	6 (1.0 %)	12 (1.4 %)
Never regular user/never used	487 (80.5 %)	647 (76.7 %)
Alcohol consumption		
Current drinker	373 (61.7 %)	315 (37.3 %)
No drinking in past year	100 (16.5 %)	221 (26.2 %)
Never drinker	132 (21.8 %)	308 (36.5 %)
BMI (kg/m^2^)^d^		
Underweight (<18.5)	182 (30.4 %)	133 (16.1 %)
Normal (18.5–24.9)	379 (63.3 %)	515 (62.2 %)
Overweight (25.0–29.9)	37 (6.2 %)	134 (16.2 %)
Obese (≥30)	1 (0.2 %)	46 (5.6 %)
Blood pressure group		
Normal	170 (28.1 %)	222 (26.3 %)
Pre-hypertension	214 (35.4 %)	299 (35.4 %)
Stage I hypertension	152 (25.1 %)	191 (22.6 %)
Stage II hypertension	69 (11.4 %)	132 (15.6 %)
HIV serostatus^e^		
Negative	554 (92.0 %)	793 (94.5 %)
Positive	48 (8.0 %)	46 (5.5 %)

^a^Missing marital status for 1 male and 1 female

^b^SES score computed from asset index based on household ownership of items within the study setting missing data for 98 males and 96 females

^c^Missing data on chewing tobacco for 1 female

^d^Missing data on BMI for 6 males and 16 females

^e^Missing data on HIV for 3 males and 5 females

**Table 2:** Haematology reference ranges [median (95th-percentile)] derived from older people in rural Uganda

Analyte (parameter)	All participants N = 903	Male N = 358	Female N = 545
Hemoglobin (g/dL)^a^	13.6 (10.9–15.8)	14.2 (10.6–16.4)	13.3 (11.0–15.0)
Haematocrit (%)^a^	40.6 (32.5–47.3)	42.2 (32.4–49.1)	39.8 (32.7–45.4)
Erythrocytes (10/L)^a^	4.63 (3.60–5.60)	4.7 (3.6–5.7)	4.6 (3.7–5.4)
Platelets (10^9^/L)^a^	220 (77–353)	207 (60–336)	229 (98–359)
WBC (×1000)	4.8 (3.0–7.7)	4.9 (3.2–7.8)	4.8 (3.0–7.2)
MCH (pg)	29.4 (25.0–33.4)	30.0 (24.7–34.2)	29.1 (25.0–32.6)
MCV (fL)	87 (75–99)	89 (73–102)	87 (76–97)
MCHC (g/L)	33.4 (32.0–35.2)	33.5 (32.0–35.2)	33.4 (32.1–35.2)
Neutrophils (no 10^9^/L)	2.05 (1.05–3.78)	2.04 (1.05–4.40)	2.05 (1.05–3.60)
Neutrophils (%)^a^	43.0 (27.2–60.3)	42.5 (26.0–62.4)	43.5 (28.1–58.5)
Lymphocytes (no 10^9^/L)	2.09 (1.15–3.51)	2.05 (1.20–3.70)	2.1 (1.1–3.4)
Lymphocytes (%)	43.9 (28.6–59.8)	43.6 (27.9–60.8)	44.2 (29.4–59.3)
Monocytes (no 10^9^/L)	0.31 (0.17–0.57)	0.33 (0.18–0.59)	0.30 (0.17–0.55)
Monocytes (%)	6.5 (4.1–10.7)	6.9 (4.3–12.1)	6.3 (3.9–10.2)
Eosinophils (no 10^9^/L)	0.15 (0.50–0.81)	0.16 (0.04–0.82)	0.14 (0.05–0.75)
Eosinophils (%)	3.2 (1–15.3)	3.4 (1.0–15.9)	3.0 (1.1–14.5)
Basophils (no 10^9^/L	0.04 (0.02–0.08)	0.04 (0.02–0.08)	0.04 (0.02–0.08)
Basophils (%)	1.0	1.0	1.0

Sample of haematology reference ranges excluding those with hookworm infection, malaria infection, HIV infection and those uncertain of their age

^a^Analytes significantly different by sex

### Haematology values for older people

The 95 % reference ranges for haematology in people aged 50 years and above are shown in Table 2. Only haemoglobin, haematocrit, erythrocyte counts white blood cells counts, platelet counts and neutrophil percent, were significantly different for men as compared to women (p < 0.05). Men had higher haemoglobin, haematocrit, erythrocyte counts and white blood cell counts than women. Women had higher platelet counts and higher neutrophils.

### Haemalotogy reference values in those below and above 65 years

The 95 % reference values for ≤65 years and those >65 according to gender are shown in Table 3. The following parameters were significantly lower in those aged above 65 years compared with those aged less than 65 years: haemoglobin, haematocrit, erythrocytes, platelets and mean corpuscular volume.

**Table 3:** Haematology reference ranges median [(95th-percentile)] comparing those above and below 65 years

Analyte	Male		Female	
	≤65 years N = 207	>65 years N = 151	≤65 years N = 321	>65 years N = 224
Hemoglobin (g/dL)^a^	14.5 (11.7–16.6)	13.6 (9.7–15.5)	13.4 (11.1–15)	13.2 (10.8–15)
Hematocrit (%)^a^	43.2 (35–49.9)	40.9 (29.6–48.1)	40 (33–45.5)	39.6 (32–45)
Erythrocytes (10/L)^a^	4.8 (3.6–5.8)	4.63 (3.5–5.8)	4.61 (3.8–5.5)	4.6 (3.6–5.3)
Platelets (10/L)^a^	220 (55–337)	201 (73–334)	222 (105–363)	235 (78–359)
WBC (10/L)	5 (3.3–7.8)	4.8 (2.9–8)	4.8 (3–7)	4.8 (3–7)
MCH (pg)	30.4 (26.1–34.2)	29.5 (23.2–33.8)	29 (24.7–32.6)	29.2 (25.6–32.7)
MCV (fl)	90 (77–102)	88 (71–100)	87 (75–99)	87 (77–97)
MCHC (g/L)	33.6 (32.2–35.4)	33.4 (31.4–35)	33.4 (32.1–35.2)	33.3 (32.1–35.3)
Neutrophils (10/L)	2.03 (1.06–4.6)	2.01 (1.03–3.8)	2.02 (1.08–3.5)	2.1 (1.04–4.1)
Neutrophils (%)	42.7 (25.8–62.2)	41.9 (27.1–63.1)	42.8 (29.5–58.2)	43.6 (27.4–59.9)
Lymphocytes (10/L)	2.13 (1.22–3.63)	1.96 (1.1–3.87)	2.11 (1.2–3.36)	2.09 (1.08–3.48)
Lymphocytes (%)	43.7 (27.8–61.2)	43.5 (27.9–60.1)	44.4 (29.8–58.8)	43.5 (28.8–68.8)
Monocytes (10/L)	0.33 (0.18–0.57)	0.33 (0.19–0.67)	0.29 (0.17–0.54)	0.31 (0.17–0.57)
Monocytes (%)	6.7 (4.3–12.1)	7 (4.5–12.2)	6.2 (3.9–10.3)	6.4 (4–10.2)
Eosinophils (10/L)	0.16 (0.5–0.8)	0.16 (0.04–0.88)	0.14 (.04–0.67)	0.13 (0.05–1.06)
Eosinophils (%)	3.4 (1–15.9)	3.4 (90.9–12.6)	3.1 (1–12.1)	2.9 (1.1–16.2)
Basophils (10/L)	0.04 (0.2–0.08)	0.03 (0.01–0.08)	0.04 (0.02–0.07)	0.04 (0.02–0.09)
Basophils (%)	1	1	1	1

^a^ Analytes significantly different in those aged 65 years and above and those below 65 years

### Comparison with haematology values in old people

We only identified one study from the African region on haematology reference ranges for old people therefore; the comparisons were from studies undertaken in high income countries. Most of the studies used different methodologies, with some reporting haematology reference ranges as 2.5th to 97.5th percentiles and some as mean ±2 SD. Table 4 shows haematology reference ranges in our study compared to other studies conducted in high income countries. All haematology parameters in our study were lower than those reported in literature [24, 36] except for a study conducted in China, where old people had MCV values that were lower than those in our study.

**Table 4:** Reference values [median (95th-percentile)] compared with those of other studies in old people

Parameter (analyte)	Current study Uganda (age 50+)	Hale et al. [26] (USA) 65+	Yip et al. [16] (USA) 65+	Tsang et al. [17] (Australia) 49+	Woo et al. [27] (China) 60+
Mean age (range)	63.5 (50–94)				
% Female	60.3				
Comorbidities excluded	Malaria, HIV, Hookworm				
Study design	Cross sectional	Cross sectional	Cross sectional	Cross sectional	Cross sectional
Hemoglobin (g/dL)					
All	13.6 (10.9–15.8)				
Male	14.2 (10.6–16.4)	−(14–18)	14.9 (12.6–17.4)	15.6 (13.1–17.5)	12.1–16.5
Female	13.3 (11–15)	−(12–16)	13.8 (11.7–16.1)	14.3 (12.2–16.1)	10.7–15
Haematocrit (%)					
All	40.6 (32.5–47.3)				
Male	42.2 (32.4–49.1)	−(42–52)	44 (37–51)	46 (39–51)	36–49
Female	39.8 (32.7–45.4)	−(37–47)	40.8 (35–47)	42 (36–47)	32–45
Erythrocytes (10/L)					
All	4.63 (3.6–5.6)				
Male	4.7 (3.6–5.7)	−(4.7–6.1)	4.82 (3.8–5.8)	5.06 (4.2–5.9)	4.0–5.7
Female	4.6 (3.7–5.4)	−(4.2–5.4)	4.5 (3.8–5.2)	4.7 (4–5.4)	3.8–5.4
Platelets (10^9^/L)					
All	220 (77–353)				
Male	207 (60–336)			240 (153–382)	95–224
Female	229 (98–359)			269 (163–414)	93–267
WBC (×1000)					
All	4.8 (3–7.7)	−(4.8–10.8)			
Male	4.9 (3.2–7.8)			6.4 (3.9–9.5)	4.6–10.5
Female	4.8 (3–7.2)			6.1 (3.6–9.4)	4.5–11.5
MCV (Fl)					
All	87 (75–99)				
Male	89 (73–102)	−(80–94)	91 (81–103)	90 (80–99)	71–96
Female	87 (76–97)	−(81–99)	90 (81–102)	90 (80–97)	70–96
Neutrophils (×10^9^/L)	2.05 (1.05–3.8)				
Neutrophils (%)	43 (27.2–60.3)				
Lymphocytes (×10^9^/l)	2.09 (1.2–3.5)				
Lymphocytes (%)	43.9 (28.6–59.8)				
Monocytes (×10^9^/L)	0.31 (0.17–0.57)				
Monocytes (%)	6.5 (4.1–10.7)				
Eosinophils (×10^9^/L)	0.15 (0.05–0.81)				
Eosinophils (%)	3.2 (1–15.3)				
Basophils (×10^9^/L)	0.04 (0.02–0.08)				
Basophils (%)	1 (0–2)				

The numbers from these four studies cited are from the original publications

## Discussion

Using blood samples drawn from older people in the anaemia survey, we established haematology reference ranges for parameters including haemoglobin, haematocrit, erythrocytes, WBC counts, platelets, mean corpuscular haemoglobin, mean corpuscular volume: number and percentages for lymphocyte, monocyte, eosinophil and basophil. We compared haematology reference values for old people aged above 65 years with those below 65 years.

To the best of our knowledge, this is the first population based study, in Uganda to describe reference values for haematology in old people. From our literature search, we only found one old study which was conducted among old Zimbabweans [20]. As research on health issues affecting older people takes root in Africa, findings from this study will be valuable in identifying old people with haematology abnormalities based on locally derived haematology reference values for old people. Compared to other studies conducted in young adults in Uganda [1, 4, 5, 15], we excluded all the study participants with malaria which has been associated with low haemoglobin, elevated white blood cell counts and thrombocytopenia [37]. We also excluded participants with hookworm infection and those with HIV infection since these have also been strongly associated with low haemoglobin values [38, 39].

Overall, old people in our study had lower haemoglobin values compared to other studies conducted in young adults in Uganda. However, old women in our study had higher haemoglobin values as compared to young adult women in Uganda and other East African countries [1, 5, 19]. The higher haemoglobin in old women compared to young adult women may be due to the fact that old women have passed through menopause and are no longer experiencing menstrual blood loss.

Old people above 65 years also had lower haemoglobin and haematocrit values as compared to those below 65 years. We do not have many studies to compare our results for people below 65 because most of the studies on haematology reference values conducted among old people in high income countries define old people as those aged 65 years and above. However, these results are in agreement with findings from the Second National Health and Nutritional Examination Survey in which haematology reference values for those aged 45–64 years tended to be higher than those aged 65–74 years [13]. Studies conducted elsewhere in high income countries have shown that haemoglobin decreases with age, with the decline more pronounced in men than women [9, 40], implying that haemoglobin reference values in old people may need to be different from haemoglobin reference values for young adults.

Haemoglobin, haematocrit, erythrocyte counts, MCV and platelet counts tended to be lower in our study than in other studies conducted among old people in high income countries [13, 14, 24, 25]. Possible explanations for these differences could be both genetic and environmental differences between Blacks and the differences in nutritional status between old whites and old blacks.

Females 65 years and older had higher platelet counts than men. Other studies have also reported these gender differences in platelet counts [4, 36].

Although we tried to exclude old people with known illnesses associated with low haemoglobin and other haematology parameters, old people who were included in this study could have had some subclinical-illnesses which we were not able to diagnose. We did not exclude smokers from our analysis even if smoking is known to elevate the total white blood cell count and neutrophils [41]. This may have not had greater impact on our findings since a small proportion of older people within the GPC smoke, especially the men. Old people in our study did not undergo fasting prior to phlebotomy. This may have affected some of the parameters. Since we bled participants between 8.30 a.m. in the morning and 2.30 p.m. in the afternoon, it was not feasible for old people to undergo fasting prior to phlebotomy.

From this study, it is clear that the haematology reference ranges based on those from high income countries commonly applied to old people in Uganda may not be appropriate for them. In addition, the haematology reference ranges for old people in our study had differences with the haematology reference ranges derived from old people from high income countries. Using appropriate haematology reference ranges for old people in Uganda will prevent the repetition and additional laboratory and clinical procedures that may be performed to old people once their haematology results do not fall under the commonly used reference ranges. In addition, it will also reduce on misclassifying old people participating in clinical trials or any other epidemiological studies as having adverse haematology abnormalities.

In conclusion, although we have described the haematology reference ranges for old people using a sample derived from a general population, there is also need for more population based studies using nationwide surveys to be carried out among old people in other study settings in Uganda and the rest of Africa to explore the differences in haematology reference values between those aged below and above 65 years of age, with a view of establishing whether there is need to have separate reference values for these different categories of old people.
